# A statistical algorithm for outbreak detection in multisite settings: an application to sick leave monitoring

**DOI:** 10.1093/bioadv/vbad079

**Published:** 2023-06-14

**Authors:** Tom Duchemin, Angela Noufaily, Mounia N Hocine

**Affiliations:** Conservatoire National des Arts et Métiers, Paris, France; Clinical Trials Unit, Warwick Medical School, Coventry, UK; Conservatoire National des Arts et Métiers, Paris, France

## Abstract

**Motivation:**

Public health authorities monitor cases of health-related problems over time using surveillance algorithms that detect unusually high increases in the number of cases, namely aberrations. Statistical aberrations signal outbreaks when further investigation reveals epidemiological significance. The increasing availability and diversity of epidemiological data and the most recent epidemic threats call for more accurate surveillance algorithms that not just detect aberration times but also detect locations. Sick leave data, for instance, can be monitored across companies to identify companies-related aberrations. In this context, we develop an extension to multisite surveillance of a routinely used aberration detection algorithm, the quasi-Poisson regression Farrington Flexible algorithm. The new algorithm consists of a negative-binomial mixed effects regression model with a random effects term for sites and a new reweighting procedure reducing the effect of past aberrations.

**Results:**

A wide range of simulations shows that, compared with Farrington Flexible, the new algorithm produces better false positive rates and similar probabilities of detecting genuine outbreaks, for case counts that exceed historical baselines by 3 SD. As expected, higher surges lead to lower false positive rates and higher probabilities of detecting true outbreaks. The new algorithm provides better detection of true outbreaks, reaching 100%, when cases exceed eight baseline standard deviations. We apply our algorithm to sick leave rates in the context of COVID-19 and find that it detects the pandemic effect. The new algorithm is easily implementable over a range of contrasting data scenarios, providing good overall performance and new perspectives for multisite surveillance.

**Availability and implementation:**

All the analyses are performed in the R statistical software using the package glmmTMB. The code for performing the analyses and for generating the simulations can be found online at the following link: https://github.com/TomDuchemin/mixed_surveillance.

**Contact:**

a.noufaily@warwick.ac.uk

## 1 Introduction

The increasing flow of data and the recent pandemic threats have necessitated development of robust epidemiological surveillance methods in multiple settings. Epidemiological surveillance is widely used for the timely monitoring of infectious disease and syndromic cases to identify sudden and unusual increases in the number of cases, more than would normally be expected, i.e. aberrations (Hulth *et al.*, 2010; Morbey *et al.*, 2015; Yoneoka *et al.*, 2021)—which might correspond to outbreaks (requiring investigation and intervention) when they are of epidemiological significance, such as influenza outbreaks (Cheng *et al.*, 2012; Duchemin *et al.*, 2021). It has also been previously used to monitor occupational health issues, such as surges of work-related injuries (Schuh *et al.*, 2014), school absenteeism or aggregated sick leave data (Airaksinen *et al.*, 2018), events related to drug and vaccine consumption (Hocine *et al.*, 2009; McNamara *et al.*, 2022), unusual concentrations of viruses in waste water and unusual amounts of Google queries (Ahmed *et al.*, 2020; Bounoure *et al.*, 2011; Kang *et al.*, 2013).

Health departments in different countries receive epidemiological data from various sources. Electronic surveillance systems and software then de-noise the data and transform it into outcome counts that get aggregated to form time series of count cases. For instance, the UK Health Security Agency syndromic surveillance team monitors daily syndromic data from five sources, the general practitioner (GP) out-of-hours and GP in-hours systems, calls to the National Health Service, emergency departments and ambulance dispatches, which report data based on the days of the week they operate. The infectious disease surveillance team receives weekly reports of confirmed cases of infections from laboratories. The diversity of such data calls for novel methods that model a variety of settings.

Epidemiological surveillance is not limited to the study of new cases over time; it has also been used to detect the location of aberrations. Most surveillance systems report both the time and location of reported cases (Unkel *et al.*, 2012), which allow the identification of aberrations by location or site too where needed. Numerous reviews of epidemiological surveillance methods have been performed (Bédubourg and Le Strat, 2017; Buckeridge *et al.*, 2005; Salmon *et al.*, 2016; Unkel *et al.*, 2012) and various statistical techniques have been used: regression, time series, statistical process control, Bayesian etc. (Unkel *et al.*, 2012). Within these frameworks, methods that would monitor simultaneously the time and location of aberrations have been used in practice (Morbey *et al.*, 2015; Shmueli and Burkom, 2010; Yamada *et al.*, 2009). These methods deal with the specifics of modelling multiple sites simultaneously over time, such as site-specific covariates adjustments and differences in variance between sites, but are known for computational complexities and their use is often limited.

In this article, we propose a statistical surveillance algorithm for multisite settings. Our algorithm is inspired by Farrington Flexible, a widely used algorithm, developed by Farrington *et al.* (1996) and improved by Noufaily *et al.* (2013), which uses a Quasi-Poisson regression model adjusted for trend and seasonality and reweighted to account for past aberrations (Hulth *et al.*, 2010). Farrington Flexible was extensively validated and evaluated in the literature. When compared to 15 other popular aberration detection algorithms (Bédubourg and Le Strat, 2017; Noufaily *et al.*, 2019), Farrington Flexible produced the highest sensitivity and specificity. We propose a multisite extension to Farrington Flexible using negative-binomial mixed effects regression, with the sites being included as a random effect in the model. This extension adds numerous levels of methodological and computational complexities to the existing model and requires methodological changes, including using negative-binomial regression instead of Quasi-Poisson, recalculating the dispersion parameter and using a different baseline reweighting threshold. The resulting algorithm can be easily implemented and can model a variety of data scenarios given by different sites. A wide range of simulations shows that compared with Farrington Flexible provides better false positive rates (FPRs) and similar probabilities of detecting genuine outbreaks. It also has a lighter computational burden than other models (Morbey *et al.*, 2015).

To our knowledge, no method was developed on the specific case of the surveillance of sick leave absences in multiple companies. In this study, we use our developed algorithm for multisite surveillance to model sick leave data across companies and monitor any possible aberrations. Companies are settings where a variety of diseases e.g. infectious diseases or stress (Duchemin *et al.*, 2019; Labriola *et al.*, 2006) can spread. These diseases could lead to spikes in the numbers of booked sick leave absences and eventually to sick leave aberrations if the absences become unusually high and if actions are not taken in time. Monitoring sick leave would help companies identify ongoing issues and hence provide a better environment for workers. Monitoring sick leave data across companies raises specific methodological issues; sick leave rates of companies show strong seasonal patterns and possible trends since they are highly correlated to seasonal infectious diseases, such as influenza (O’Reilly and Stevens, 2002). Moreover, sick leave rates are associated with exogenous events, such as school holidays and the population of workers in each company (age is for instance associated with sick leave rates) (Airaksinen *et al.*, 2018).

This article develops an algorithm for multisite epidemiological surveillance using sick leave across companies as a case study. Section 2 describes the developed mixed effects regression algorithm. Section 3 presents the simulation study run to investigate the algorithm’s outbreak detection performance. Section 4 displays exploratory analyses to investigate the optimal reweighting threshold for our algorithm for different outbreak sizes. In Section 5, we present an application to sick leave data in the context of COVID-19. Finally, we discuss our findings in Section 6.

## 2 The algorithm for aberration detection in multisite settings

The algorithm is an adaptation of Farrington Flexible (Noufaily *et al.*, 2013) to a multisite setting. Farrington Flexible outputs an aberration threshold based on a Quasi-Poisson regression adjusted for trend and seasonality and reweighted to reduce the effect of previous outbreaks. Inspired by Farrington Flexible, the new algorithm fits a negative-binomial mixed effects regression model.

### 2.1 The new algorithm steps

The algorithm monitors the weekly number or counts of cases (e.g. epidemiological cases) simultaneously over time and across different sites. To determine if the number of cases, *Y_i, T,_* in a site *i* ∈ {1*,…,N*} with *N* *>* 0 at week *T* *>* 0 is higher than a pre-specified alert threshold, the new algorithm uses three steps:

Firstly, we fit a negative-binomial regression on the past counts *Y_i,t_* with *t* ∈ {0*,…,T*} adjusting for trend, seasonality, covariates and a random effect, *u_i_* ∼*N*(0*,σ*^2^), representing the sites’ effects: where μ*_i,t_* is the mean of the negative-binomial distribution and *θ* is the dispersion parameter such that $X_{i,t\;}$is a matrix of covariates and *β* are the corresponding parameters. *δ_t,T_* is a matrix of level factors adjusting for seasonality and giving more weights to the comparable periods (to *T*) in past years, similar to Farrington Flexible (Noufaily *et al.*, 2013). Each column of *δ_t,T_* describes a period: a first reference 7-week period (corresponding to weeks *T* ± 3 weeks) and nine 5-week periods in each year. It is thus a matrix with 10 columns (one per period) with 1 indicating the associated period and 0 otherwise; *γ* are the corresponding parameters. To avoid the effect of emerging outbreaks on the model, we exclude the 26 most recent weeks from the baseline data and we only fit the regression model on the previous weeks.


$$Y_{i,t}∼\mathrm{NB}\;\left(\mu_{i,t},\;\theta \right),\;\mu_{i,t}=\exp\left(\beta_{0}+\beta X_{i,t}+\gamma \delta_{t,T}+\nu t+u_{i}\right),$$

$$\mathrm{Var}\left(Y_{i,t}\right)=\mu_{i,t}+\frac{\mu_{i,t}^{2}}{\theta },$$

Secondly, we down-weight the effect of baseline aberrations to fit a more robust aberration threshold. We use the following weight function. where *S* *>* 0 is a constant controlling the severity of reweighting and *r_i,t_* are the Pearson residuals defined as with $\hat{\mu_{i,t}}\;$and $\hat{\theta }\;$the respective estimates of *μ_i,t_* and *θ* from the fitted model.


wi,t =  ωSri,t if ri,t>Sω otherwise s.t. ∑i,twi,t = NT, ∀ i,t >0 

$$r_{i,t}=\frac{Y_{i,t}-\hat{\mu_{i,t}}}{\sqrt{\hat{\mu_{i,t}}+{\hat{\mu_{i,t}}}^{2}/\hat{\theta }}}\;\forall i,t>0,$$

Finally, we compute the aberration threshold. We fit a new negative-binomial regression model to our data using the previous reweighting procedure. The aberration threshold of site *i* at time *t* is defined as where $Q_{\hat{\mu_{i,t}},\hat{\theta }}$ is the quantile function of a negative-binomial distribution, with $\hat{\mu_{i,t}}\;$and $\hat{\theta }\;$the respective estimates of *μ_i,t_* and *θ* obtained from the second regression model, and *α* the type I error.


$$U_{i,t}=Q_{\hat{\mu_{i,t}},\hat{\theta }}\left(1-\frac{1-\alpha }{2}\right),$$

All the analyses are performed in the R statistical software (R Development Core Team, 2023) using the package glmmTMB (Brooks *et al.*, 2017).

### 2.2 Comparison with Farrington Flexible

Compared to Farrington Flexible, the new multisite algorithm has five key differences:

1. It fits a negative-binomial regression model instead of a Quasi-Poisson since the former provide a more natural and easily implementable mixed effects model for over-dispersed data. We faced computational complexities fitting the Quasi-Poisson mixed effects model.
2. It adjusts for the effects of company or site-related covariates, such as the population demographics within each site. This is an important step since it allows better modelling of the site effects.
3. The main change is the inclusion of a random effects term for the site. This resulted in the modification of the algorithm since the individual outcome is now calculated simultaneously at both the time and site levels.
4. It recalculates the dispersion parameter based on the new negative-binomial mixed effects model using maximum likelihood estimation and Laplace estimation via the R function glmmTMB (Brooks *et al.*, 2017).
5. It down-weights past aberrations using the standardized Pearson residuals instead of the standardized Anscombe residuals since the latter are not straightforward to calculate in a mixed model framework.

## 3 Simulation study

In this section, we investigate the validity of our model using an extensive simulation study similarly to Noufaily *et al.* (2013, 2019) to allow for a comparison with those studies.

### 3.1 Simulated datasets and outbreaks

#### 3.1.1 Baseline data 

We generate the baseline data using a Negative-Binomial model of mean

*μ* and variance $\mu +\frac{\mu^{2}}{\theta }$ with *θ* *>* 0 the dispersion parameter. To be consistent with Noufaily *et al.* (2013), we reparametrize the negative-binomial distribution such that its variance equals to ϕμ,ϕ being the new dispersion parameter, by reparametrizing *θ* as:


$$\theta =\frac{\bar{\mu }}{\phi -1\;}\;\mathrm{with}\;\bar{\mu }=\frac{1}{it}\;\sum_{it}\mu_{it}.$$

At week *t* ∈ {1*,…,T*} and for site *i* ∈ {1*,…,N*}, we define *μ_i,t_* as: where *β*_0_ represents the volume of the outcomes, $\beta_{X}$ and $\beta_{Z}$ are the respective coefficients of the two covariates *X_i,t_* and *Z_i,t_*, which we define below, and ν corresponds to the trend. The Fourier term represents seasonality and finally *u_i_* ∼*N*(0*,σ*^2^) with *σ* *>* 0 is the random effect of the different sites. In practice, we expect to have both continuous and discrete covariates. The covariates should be stable within each site but could be very different from one site to another. For site *i*, we generate $X_{it}$ and $Z_{it}$ vectors of length *t* as the following: $X_{it}∼N\left(m_{i},1\right)\;$with $m_{i}∼U\left(30,50\right)$ and $Z_{it}∼\mathrm{Bernoulli}\left(p_{i}\right)$ with $p_{i}∼U(0,1)$.


$$\mu_{i,t}=\exp\left(\beta_{0}+\beta_{X}X_{it}+\beta_{Z}Z_{it}+\nu t+\sum_{s=1}^{2}\gamma \left(\cos\left(\frac{2\pi st}{52}\right)+\sin\left(\frac{2\pi st}{52}\right)\right)+u_{i}\right),$$

In all the simulations, we set *N* = 50 sites and *T* = 312 weeks corresponding to 6 years of data. The most recent 52 weeks constitute the current dataset for which we evaluate the model and the previous 260 weeks constitute the baseline data. We also fix the reweighting threshold to *S* = 2.5 and the type I error to *α* = 0.05.

#### 3.1.2 Outbreaks

We simulate (the effects of) outbreaks as the following:

1. Outbreak start weeks: We randomly select 4 weeks from the baseline data and 1 week from the current data. These will be the starting weeks of four baseline outbreaks and one current outbreak.
2. Outbreak sizes: For each selected outbreak start week *t*_0_ *>* 0 from the baseline negative-binomial data, we compute the mean $\mu (t_{0})\;\mathrm{and}\;\mathrm{the}\;\mathrm{standard}\;\mathrm{deviation}\;\mathrm{SD}(t_{0})\;=\;\sqrt{\mu (t_{0})\phi \;}$. We then randomly generate one value from a Poisson distribution of mean equal to *k* *>* 0 times SD(*t*_0_) and we set this value to be the outbreak size *q*_0_. In this article, we consider *k* * = * 1–2 as representing a small outbreak, *k* * = * 3–5 as a medium outbreak and *k* * = * 8–10 as a large outbreak.
3. Outbreak distribution: For each selected outbreak start week *t*_0_ *>* 0 and generated outbreak size *q*_0_, we inject *q*_0_ lognormally distributed data points of mean 0 and SD 0.5. For each week *t*_0_ *>* 0, we finally distribute these lognormally distributed points starting from *t*_0._

### 3.2 Simulation scenarios

To evaluate the robustness of the model to a wide range of data sets encountered in real life, we generate simulations from 32 parameter combinations as described in Table 1. We generate different: baseline volumes (given by *β*_0_), trends (given by ν), covariates (given by *β_X_* and *β_Z_*), overdispersions (given by ϕ) and standard deviations for the random effect (given by *σ*^2^). We set the *γ* parameter for seasonality to 0.02. For each of the 32 simulation scenarios, we perform five replications of *N* = 50 sites for *T* = 52 current weeks. We perform only five replications because the computation is time-consuming. The code to generate the simulations used in this study can be obtained online from the link: https://github.com/TomDuchemin/mixed_surveillance.

**Table 1.** Parameter values for replicating the 32 simulation scenarios

Scenario	*β*_0_	ν	*Β_X_*	*β_Z_*	*Φ*	*σ* ^2^
1	1	0	0	0	1.5	0.5
2	1	0	0	0	1.5	1.5
3	1	0.0025	0	0	1.5	0.5
4	1	0.0025	0	0	1.5	1.5
5	1	0	−0.5	1	1.5	0.5
6	1	0	0.5	1	1.5	1.5
7	1	0.0075	−0.5	0.5	1.5	0.5
8	1	0.0075	−0.5	0.5	1.5	1.8
9	3	0	0	0	1.5	0.5
10	3	0	0	0	1.5	2
11	3	0.0025	0	0	1.5	0.5
12	3	0.0025	0	0	1.5	2
13	2	0.0025	−1	1	1.5	0.5
14	2	0.0025	−1	1	1.5	1
15	2	0.0075	−0.5	0.5	1.5	0.5
16	2	0.0075	−0.5	0.5	1.5	1.8
17	1.5	0	0	0	3	0.5
18	1.5	0	0	0	3	1.5
19	1.5	0.0025	0	0	3	0.5
20	1.5	0.0025	0	0	3	1.5
21	0.5	0.0025	−1.5	1.5	3	0.5
22	0.5	0.0025	−1.2	1.2	3	1.5
23	0.5	0.0075	−0.5	0.5	3	0.5
24	0.5	0.0075	−0.5	0.5	3	1.5
25	3	0	0	0	3	0.5
26	3	0	0	0	3	1.5
27	3	0.0025	0	0	3	0.5
28	3	0.0025	0	0	3	1.5
29	3	0.0025	−1.2	1.2	3	0.5
30	2	0.0025	−1.2	1.2	3	1.5
31	3	0.0075	−0.5	0.5	3	0.5
32	2	0.0075	−0.5	0.5	3	1.5

### 3.3 Evaluation metrics

We evaluate the performance of our model using two metrics. The first metric is the FPR, the probability of a false alert, which we calculate as the proportion of observations where the observed value exceeded the threshold in the absence of a current outbreak. The FPR is a rate per week. The second metric is the Probability of Detection (POD), the probability of a true alert, which is calculated as the proportion of observations where the observed value exceeded the threshold at least 1 week in the presence of a current outbreak. POD is defined as the proportion of true alerts detected among the 50 companies in each replicate.

We run both, the new mixed effects algorithm and Farrington Flexible, on five replicates of each of the 32 scenarios in Table 1 (the most recent 52 weeks) from each of 50 companies. Hence, we run 416 000 simulations in total. We first perform the simulations for a fixed medium baseline and current outbreak size (*k* = 3 SD) and reweighting threshold *S* = 2.5. For each of the five replicates of the simulations, we compute both evaluation metrics, FPR and POD. In the next section, we undertake exploratory analyses to investigate the most appropriate value for *S*.

### 3.4 Simulation results

#### 3.4.1 False positive rates

**Figure 1.**
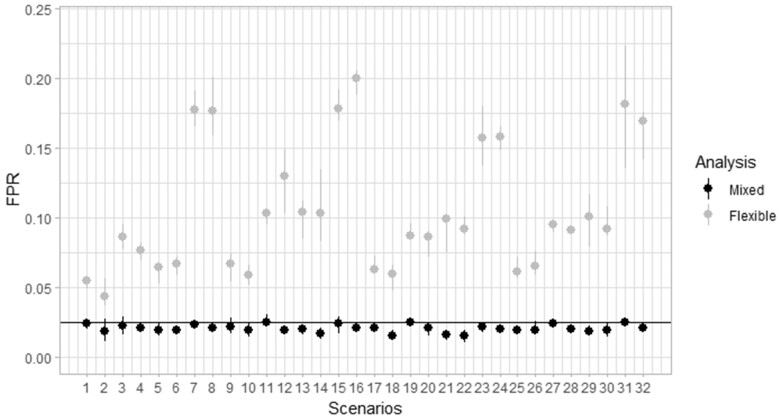
FPRs from both, the new mixed effects model and Farrington Flexible, across the 32 scenarios for *α* = 0.05, *s* = 2.58 and *k* = 3 SD. Each point represents the median FPR for the five replicates, 52 current weeks and 50 companies. The horizontal line represents the nominal value of 0.025

Figure 1 shows the FPRs from both, the new mixed effects model and Farrington Flexible, for each of the 32 scenarios, with set *α* = 0.05, medium outbreak size (*k* = 3) and reweighting threshold *S* = 2.5. For each replicate within each scenario, we calculated the mean FPR of the 52 current weeks across 50 companies. Each point represents the median FPR of the five replicates within each scenario. The vertical line represents the range of FPRs for those five replicates. The nominal FPR is 0.025. We observe that, for the new algorithm, the calculated FPRs are very slightly lower than 0.025. Scenarios with no trend and with low random effect present higher FPRs but still lower than 0.025 in median. For all scenarios, the new algorithm produces much better FPR (closer to 0.025) than Farrington Flexible.

#### 3.4.2 Probability of detection

**Figure 2.**
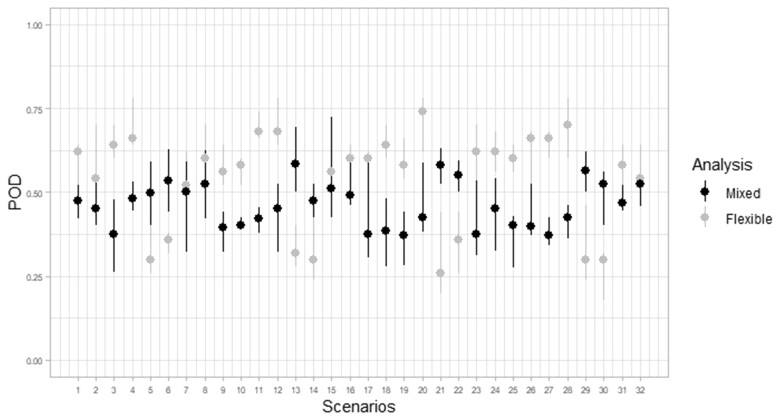
POD from both, the new mixed effects model and Farrington Flexible, for each of the 32 scenarios for *α* = 0.05, *S* = 2.5 and *k* = 3 SD. Each point represents the median POD for the five replicates, 52 current weeks and 50 companies

Figure 2 shows the PODs from both, the new mixed effects model and Farrington Flexible, for each of the 32 scenarios, for set *α* = 0.05, medium outbreak size (*k* = 3) and reweighting threshold *S* = 2.5. Each point represents the median POD of the five replicates across the 52 current weeks and 50 companies. The vertical line represents the range of PODs for the five replicates. PODs from both methods vary around 0.5. The new mixed effects method gives, in general, higher values for scenarios with covariates.

## 4 Exploratory analyses

For computational reasons, the previous simulations fixed the values of some parameters, such as *S* and *k*, which may have an impact on the results of the model. To investigate the impact of those parameters, we performed exploratory analyses using the seventh simulation scenario (from Table 1) whose parameter values all have a medium effect on the model.

### 4.1 Reweighting threshold

**Figure 3.**
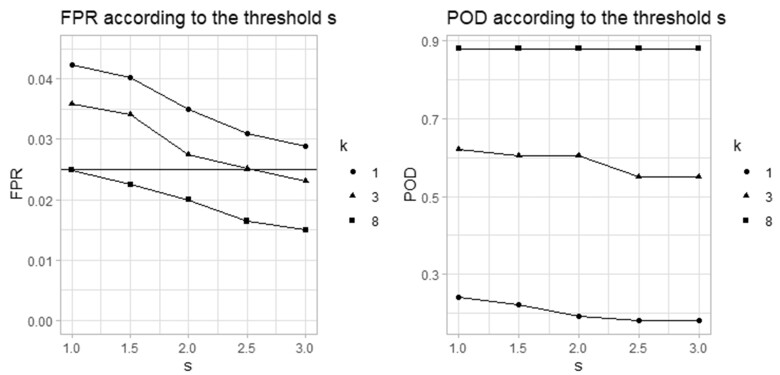
Simulation scenario seven FPR and POD from the new mixed effects model for different values of the reweighting threshold *S* and outbreak sizes *k* = 1, 3 and 8 SD

Figure 3 shows the FPRs and PODs from the new mixed effects algorithm, obtained for different reweighting thresholds *S* = 1, 1.5, 2, 2.5 and 3, for medium outbreak size (*k* = 3) and *α* = 0.05. As expected, we find that a higher threshold leads to lower FPR; the underweighting is less strict, which leads to less alerts. The optimal *S*, which is the one that gives an FPR around the nominal value, is different according to the outbreak size.

For large outbreaks (*k* = 8), we should choose a low *S* around 1. For medium outbreaks (*k* * = * 3), we should choose a medium *S* around 2.5 (which is the value we used for our simulations above). For small outbreaks (*k* * = * 1), a higher *S* should be chosen (*S* = 3). The results are consistent with Noufaily *et al.* (2013) who suggested that the optimal value for *S* is 2.58. The POD remains almost constant for all values of *S*. The choice of the threshold *S* will mostly influence the FPR and hence the specificity of the model.

### 4.2 Outbreak size

**Figure 4.**
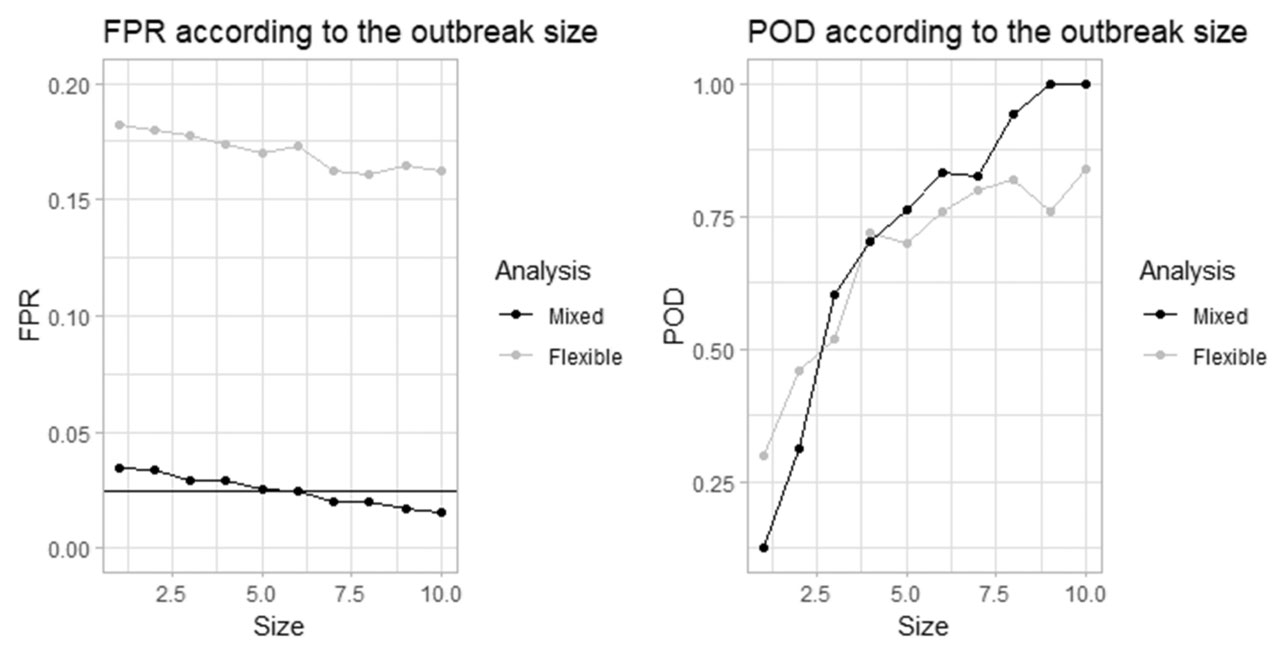
Simulation scenario seven FPR and POD from both, the new mixed effects model and Farrington Flexible, for different outbreak sizes *k* (from 1 to 10 SD) and fixed reweighting threshold *S* = 2.5

Figure 4 shows the FPRs and PODs from both, the new mixed effects model and Farrington Flexible, obtained for different outbreak sizes *k* (from 1 to 10) with *S* = 2.5 and *α* = 0.05. The same baseline dataset is used for the different *k*’s (only the outbreaks are modified) to isolate the impact of the outbreak size. FPRs are lower when the outbreak size is higher, which underlines yet again that it is preferable to adjust the reweighting threshold *S* according to the expected size of outbreaks. On the other hand, the POD increases greatly with the value of *k.* The new mixed effects method FPR is around the nominal value of 0.025 whereas Farrington Flexible FPR is much higher, around 0.17. The new mixed effects method POD becomes higher than the Farrington Flexible POD for larger outbreaks (from *k* = 3 SD), approaching almost 100% after *k* = 8.

## 5 Case study: sick leave monitoring and the example of COVID-19

### 5.1 Data

The dataset, named Declaration Sociale Nominative, reports daily sociodemographic characteristics, administrative statuses and sick leave absences of all employees from January 2018 to May 2020 of 1376 French companies (which have more than 50 employees) insured by Malakoff Humanis, a French health insurer. Our study data are reconstructed here to obtain one line per week for each company: week describing the total number of patients on sick leave, the total number of employees, as well as sociodemographic variables.

For each company, we monitor the total number of sick leaves booked on a weekly basis between January 2018 and May 2020. The model outcome is the weekly total of booked sick leave days for each company. The total number of theoretical working days per week is included in the model as an offset to adjust for the company size. The dataset also describes some characteristics of the companies, which we include in the model as covariates: the number of employees per category of age (35 years old and less, 36–45 years old, 46–55 years old, 56 years old and more), the number of workers with a temporary contract and the number of workers per occupational categories (managerial occupations, intermediate occupations, manual lower occupations and non-manual lower occupations). We also add an indicator for weeks with high numbers of vacation days that correspond to low levels of sick leaves. A report from the statistics department of the French Ministry of Labour (DARES, 2017) shows that peaks in annual leave occur during the Christmas school holidays (last week of December and first week of January) and during the summer (second week of July to third week of August).

Our model’s baseline data are from 2018 and 2019 and our current data to which we run the model are from January to May 2020. We set the type I error to *α* = 0.05 and the reweighting threshold to *S* = 2.5.

### 5.2 Results

**Figure 5.**
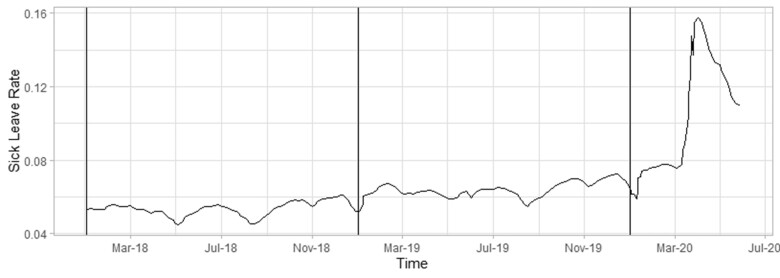
Weekly mean sick leave rates among all companies

Figure 5 shows the evolution of the mean sick leave rate from January 2018 to May 2020. We observe a much higher rate in 2020 because of the COVID-19 pandemic. Before the third week of March 2020, the sick leave rate follows a distribution that is similar to the previous years. A peak is observed at the third week of March 2020 and corresponds to the first week of lockdown in France. This high sick leave rate should not be interpreted as a high incidence of COVID-19 patients but as an implication of regulatory change: employees who had to stay home with their children were provided sick leave.

We run the new mixed effects algorithm on the 2020 sick leave data to identify companies, which were impacted by COVID-19 after the lockdown and companies, which had alerts non-related to COVID-19 before lockdown. When the total number of sick leaves on a certain week exceeds the alert threshold on that week, an alert is signalled. Table 2 displays the results of the algorithm run on the dataset in 2020. We observe that, before lockdown, 5.9% and 7.9% of the companies have exceeded the alert threshold in January and February, respectively. After March, the number of companies with alerts rises to 56.8% in March, 58.9% in April and 42.1% in May. Therefore, more than half of the companies seem to have been affected by COVID-19 in terms of sick leave. The companies with an alert have higher sick leave rates after lockdown (17.7% in April and May) than before (8.3% in January and February).

**Table 2.** Proportion of companies with a sick leave alert using the new algorithm and sick leave rates in the first 5 months of 2020

	January (%)	February (%)	March (%)	April (%)	May (%)
Proportion of companies with an alert	5.9	7.9	56.8	58.9	42.1
Sick leave rate among companies with no alerts	7.5	7.6	9.6	8.0	6.5
Sick leave rate among companies with alerts	8.3	8.3	14.4	17.7	17.7

**Figure 6.**
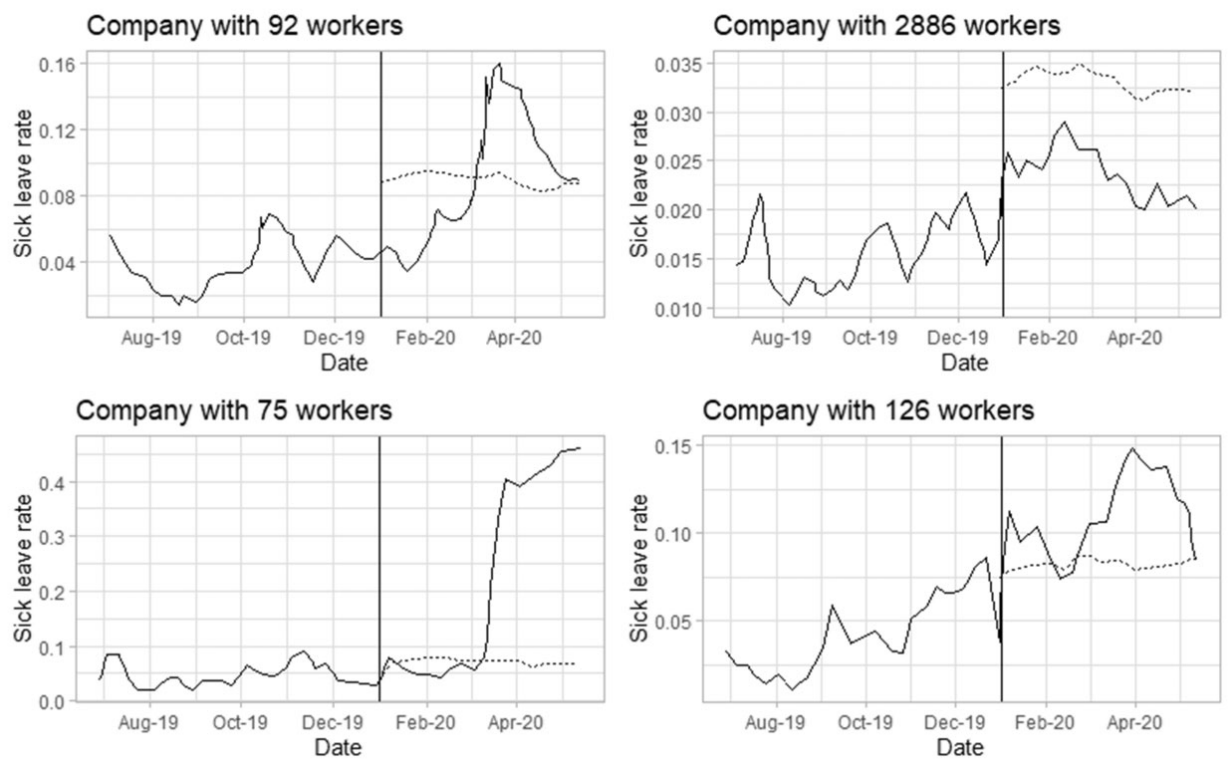
Four examples of companies’ sick leave rates from July 2019 to May 2020. The vertical line represents the first day of 2020, the solid line represents the weekly sick leave rate of the company and the dashed line represents the alert threshold from the new mixed effects algorithm

Figure 6 shows four selected examples of companies’ sick leave rates with different numbers of employees and alerts signalled by the new algorithm. The first company represents a case where the alert threshold is exceeded just after lockdown and then the sick leave level goes back to the baseline level. The second company presents no alerts. The third company highly exceeds the alert threshold just after the lockdown and the sick leave level stays very high. The fourth company exceeds the alert threshold at the beginning of the year. The solid line in Fig. 6 represents the weekly sick leave rate of the company and the dashed line is the alert threshold. We observe that for each company, the alert threshold is consistent with the baseline level of sick leave and has not been majorly affected by the pandemic. It is difficult to identify the genuine outbreaks in our sick leave data and therefore to compute and compare FPRs and PODs. The aim here is to check that the new model alerts due to sick leave surges around lockdown. The results are reassuring since they show that the new algorithm identifies the sudden increases in sick leaves. This is also the case with Farrington Flexible. If the increase in sick leaves becomes permanent then, with time, the increased level would become the new baseline and the alert threshold would adjust to this change.

## 6 Discussion

We presented an extension to the Farrington Flexible algorithm for the surveillance of multisite data, and we proposed an application to the case of sick leave in multiple companies. Modelling count outcome as functions of both time and site required an extra dimension (the site level) in the existing model. The addition of a random effect for the influence of sites allowed the simultaneous monitoring of aberrations over time and across sites, and resulted in changing the choice of the weight function and of the alert threshold. Each site is different and has its own rules and demographics. The addition of site-specific covariates provided a better modelling of the different sites and a truer representation of authentic data scenarios. Extensive simulations showed that for medium outbreaks (*k* = 3 SD), the new mixed effects algorithm provides better FPR than the existing Farrington Flexible and similar POD. We also found that, for medium outbreaks, the optimal reweighting threshold *S* was 2.5 and it led to average FPR between 0.015 and 0.025 and POD between 0.368 and 0.616. As expected, higher outbreak sizes led to higher POD and lower FPR. For larger outbreaks, Farrington Flexible provided better POD, reaching almost 100% when *k* is larger than 8 SD.

Computation time could be an obstacle for mixed effect models (Morbey *et al.*, 2015). Approximately 1 h and a half was needed to run our algorithm for 1376 companies. To improve this computational complexity, the model could be stratified by groups of companies (by company size for instance) run in parallel. Our model is not necessarily the most appropriate one for all situations but is certainly of need in particular multisite settings where each site has its own specifications. We have seen that in the presence of covariate effects, the new algorithm could perform particularly well in comparison with Farrington Flexible.

Mixed model surveillance is already used in practice to monitor syndromic aberrations (Morbey *et al.*, 2015). Our extension of the Farrington algorithm to a multisite setting is applicable to a range of contrasting data scenarios, is accurate and can provide better sensitivity since its baseline data are modelled using level factors and reweighted to account for past aberrations. It also allows the addition of covariates and specifications as needed, such as the addition of random effects on the time trend e.g. and the selection of model parameters based on simulations.

The application to sick leave provides interesting results in the case of COVID-19 and helps identify companies that were impacted by the pandemic. We did not use 5 years of historical data as suggested by Noufaily *et al.* (2013) because our data did not go back as far in time. However, we believe that this model can be fitted on a smaller time window since this is compensated by the large number of companies. In practice, this surveillance system could help identify and alert companies that have unusual levels of sick leave in a timely manner to monitor potential issues. A sick leave alert can, e.g. be related to issues within the company or administrative problems. Investigations should be done in order to identify the reasons behind the unusual increase in sick leaves, which could be disease-related, company-related, due to exogenous factors, such as holidays and media hypes etc. This study provides a validated surveillance algorithm that can be used in practice in multisite settings such as surveillance across geographical locations, areas and companies.

## Data Availability

The data that support the findings of this study are available from Malakoff Humanis but restrictions apply to the availability of these data, which were used under license for the current study, and so are not publicly available. Data are however available from the authors upon reasonable request and with permission of Malakoff Humanis.
